# The Potential of Hormonal Contraception to Influence Female Sexuality

**DOI:** 10.1155/2019/9701384

**Published:** 2019-03-03

**Authors:** Filipa de Castro Coelho, Cremilda Barros

**Affiliations:** Department of Gynecology and Obstetrics, Hospital Dr. Nélio Mendonça, Serviço de Saúde da Região Autónoma da Madeira, E.P.E., Funchal, Portugal

## Abstract

The association between female sexual function and hormonal contraception is controversial. Recognition and management of sexual side effects in women using hormonal contraceptives are challenging. An unsatisfactory number of studies report the influence of the available contraceptives on female sexuality. This article provides an updated narrative review regarding the effect and the magnitude of the impact that hormonal contraceptives play in female sexual function.

## 1. Introduction

Female sexual dysfunction (FSD) takes different forms, including lack of sexual desire, impaired arousal, inability to achieve orgasm, or pain with sexual activity. It can be defined as a persistent or recurrent sexual problem that causes marked distress or interpersonal difficulty [1]. It may be a lifelong problem or acquired after a period of normal sexual function.

Female sexual problems are highly prevalent worldwide. The prevalence of FSD ranges from 27% to 70% [2–4]. A population-based study with women aged 18 to 79 years found that 37.9% of the Portuguese women report symptoms regarding sexual health [5]. A similar percentage of women have sexual concerns in the United States [6].

The etiology of FSD is often multifactorial and may include psychological problems such as depression or anxiety, relationship conflict, fatigue, stress, issues relating to prior physical or sexual abuse, medications, and physical problems such as endometriosis or genitourinary syndrome of menopause [7]. Female sexual function is influenced by several biological, psychological, and social factors.

The general framework sequence of the female sexual response cycle is described in four phases: desire (libido), arousal (excitement), orgasm, and resolution [1, 7]. However, this cycle of responses must be understood within an intrapersonal and interpersonal context. As previously mentioned, factors as depression or the length of relationship have been associated with FSD [8, 9]. For many women, the contemporary phases of the sexual response cycle may vary in sequence, overlap, repeat, or even be absent, during all or some sexual encounters [7, 8].

In fact, a women's subjective satisfaction with the sexual experience may not require achieving all response phases. The solid correlation observed in men, between subjective arousal and genital congestion (erection), is not seen in women. Most studies identify little correlation between a woman's complaints of limited genital arousal and objective measures of vulvovaginal blood flow and engorgement. Arousal may relate to physiological phenomena as vasocongestion and genital lubrication, and/or to thoughts and feelings. Self-reports by women often do not distinguish between desire and arousal. In addition, spontaneous desire is unusual in women, except in new relationships, which emphasizes the fact that the absence of this domain does not necessarily mean a disorder [7, 10].

Low sexual desire is the most commonly reported female sexual health problem [5, 6]. Significant increases in libido and in sexual frequency were seen in postmenopausal women who received weekly intramuscular injections of testosterone [11]. Sexual desire was significantly associated with levels of free testosterone (FT) and androstenedione in premenopausal women, but FSD in general was not associated with androgen levels [9]. A population-based study of over 1400 randomly selected women aged 18 to 75 years showed no single androgen level to be predictive of low sexual function [12].

The female sexual response is complex. The assumption that androgen levels are a key factor in female sexual function is based on the role of androgens in male sexuality. Although exogenous androgens administration exerted a positive balance on some aspects of female sexuality, in such studies, however, the levels of circulating testosterone achieved have been supraphysiological [11, 13, 14].

The biological activity of circulating sex steroids is dependent upon the relative binding to sex hormone-binding globulin (SHBG). The unbound fraction or FT is biologically active. 98 percent of circulating testosterone is protein bound, mainly to SHBG or albumin. Binding to albumin is weak, so this hormone portion may also be bioavailable [15, 16]. Apart from the cyclical variation in ovarian androgens, there is not the same homeostatic regulation of FT levels in women as in men, and there is a greater variety of factors that are likely to affect steroid binding in women compared to men (e.g., exogenous estrogen) [14, 17].

Hypoestrogenism results in signs and symptoms with impact on sexual function: reduction of vulvovaginal lubrication, vasocongestion during sexual arousal, vaginal atrophy, and dyspareunia [18, 19]. But factors that increase SHBG concentrations, as estrogen therapy, pregnancy, or oral contraceptives (OCs), will decrease FT. However, there is a lack of evidence that any impact of OCs on sexual function is caused by decreased androgen activity. And, above all, there are conflicting data about hormonal contraceptives as a risk factor for FSD. The relationship between female sexual function and hormonal contraception is controversial.

This is a narrative review of existing data that explores the effect and the magnitude of the impact that hormonal contraceptives play in female sexual function.

## 2. Contraception and Sexuality

In 1914, a feminist nurse named Margaret Sanger started believing that women could have reliable contraception. In 1960, “the pill” became reality as a contraceptive approved by Food and Drug Administration (FDA). Soon, couples had the right to engage in nonprocreative sex and the choice of having children and when to have them.

Today, hormonal contraception is in general an affordable, reversible, and highly effective form of family planning, available with different components (various progestins and/or estrogens), and with different routes of administration. Again, this panoply of possible options reinforces the right to choose. The woman gained control with these methods of contraception: control over methods (contrary to the male condom) and control of sexual availability (despite the number of days from last menses). The frequency of sexual activity and sexual enjoyment correlate positively with contraceptive satisfaction [20]. Fear about an unwanted pregnancy was associated with a negative impact on women's sexual arousal, particularly if one's partner did not share the same concern. Although some men often do not consider their responsibility to think about contraception, some women feel that a partner's shared concern could serve to buffer potential negative effects on their ability to feel aroused [21].

Hormonal contraceptives were not thought to improve sexual function, in addition to the reduction in concern about the risk of an unplanned pregnancy. However, female contraception has changed sexuality. An unsatisfactory number of studies report the impact of the available contraceptives on female sexuality. This is true even for combined oral contraceptives (COCs) introduced half a century ago. Even so, there are potential consequences for the female sexual response linked to the various contraceptive methods currently in use, especially in the case of hormonal contraception. Women's choice for sexual activity without pregnancy should also be balanced with the plausible risks and benefits of contraception for female sexual function.

Sociocultural barriers, taboos, and misconceptions make the assessment of female sexuality difficult. Clinical, social, and relationship characteristics of the couple may also tamper a correct evaluation of the etiology of sexual dysfunction. The possible sexual side effects of hormonal contraceptives are unknown to many patients and are dubious to many physicians. Challenges in initiating communication on sexual health continue to be present in clinical practice [22]. However, it is important that the influence of hormonal methods upon female sexual function be discussed with women during contraceptive counseling. Otherwise, omission of the sexual issues would be counter to the original intent of “the pill” and therefore could affect the reproductive and sexual health of users of hormonal contraception.

## 3. Hormonal Contraceptives and Female Sexual Function

The association between hormonal contraception and sexuality is an important issue of debate and study. Although there is currently no recommendation to screen for sexual dysfunction before or during the use of hormonal contraception, various contraceptive methods have been associated with changes in women's sexual health. In general, there are mixed effects on several sexual domains caused by hormonal contraceptives. There is no consistent pattern regarding route of administration or estrogen-progestin versu*s* progestin-only contraceptives.

### 3.1. Progestin Androgenicity and Estrogen Dose

The endocrinology of female sexual function is uncertain. COCs supress ovarian testosterone levels via suppression of pituitary luteinizing hormone (LH). The estrogen component of the pill is metabolized in the liver and increases hepatic production of SHBG, with a consequent decrease in FT concentrations. Some progestins decrease SHGB levels, but the overall effect of COCs is an increase in SHBG levels. On a scale vision, all COCs are antiandrogenic, although some are more than others depending on the progestin component [14, 17, 23].

Other hormonal methods can affect SHBG levels as well. The combined contraceptives, patch and vaginal ring, have both been found to increase serum SHBG levels even more than COCs [24–26].

Also, while a lower ethinylestradiol (EE) contraceptive pill reduces FT less, higher doses of estrogen do not appear to increase the likelihood of sexual side effects [27–29]. Indeed, a prospective randomized study observed the highest rates of negative impact on sexual well being reported by COC 15 mcg EE users versus 20 mcg EE users [29]. Another study randomized 97 women to either 30 mcg EE/150 mcg levonorgestrel (LNG) or 20 mcg EE/100 mcg LNG. Both groups showed improvements in sexual function, but this was only with statistical significance for the lower dose group. The highest dose group decreased plasma androgen levels, but with no impact on sexual desire. In addition, sexual desire increased among users of the lower dose formulation [30].

As previously described, it has been proposed that androgens play a role in female sexual function, but correlations between indices of androgenicity and the various measures of sexuality are inconsistent. A double blind randomized control trial, comparing effect of drospirenone (DRSP) [3 mg + 30 mcg EE] and gestodene (75 mcg + 20 mcg EE), revealed significant improvements regarding sexual desire, arousal, and overall satisfaction in the DRSP group. An increased frequency of orgasms in the gestodene group was reported. And the diagnosis of FSD and the free androgen index showed no statistically significant differences between the two treatments [27]. Both OCs formulations, one with an antiandrogenic activity, the other a non-anti-androgenic progestin, were not associated with negative sexual effects. The relevance of changes in androgen levels in female sexual function remains unclear. It seems plausible that some women may be more susceptible to androgen level alteration than others.

### 3.2. Short-Acting Reversible Contraception

Long-acting reversible contraception (LARC) consists of intrauterine devices (IUDs) and subdermal implants; all other reversible methods provide shorter-term protection and can be categorized as short-acting reversible contraception (SARC).

#### 3.2.1. Oral Contraceptives

OCs can be divided into two main groups: COCs with estrogen and progestin components and progestin-only pills (POPs). Contraceptive action is provided by the inhibition of the hypothalamic-pituitary-ovarian (HPO) axis, suppressing follicular growth and inhibiting ovulation. The essential component for any hormonal contraceptive method is the progestogen. The primary role of this component is to prevent ovulation through a negative feedback mechanism that results in a decrease in LH. Progestogen action also reduces the receptivity of cervical mucus and decreases endometrial thickness [31, 32]. Estrogens contribute to the contraceptive mechanism by inhibiting both follicle-stimulating hormone (FSH) and LH. Estrogen provides better regulation of the HPO axis. The inhibition of FSH seems to be related to estrogen dose, because more follicular activity is seen with progestogen-only methods than with COCs [33, 34].

*(1) Combined Oral Contraceptives*. COCs remain one of the most commonly used forms of family planning. The majority contain EE as the estrogen component. COCs formulated to include estradiol (E2) have recently become available for the indication of pregnancy prevention. A combined estradiol valerate and dienogest pill (E2V/DNG) and a COC containing 17*β*estradiol and nomegestrol acetate (E2/NOMAC) are the newest alternatives to the first synthetic estrogen.

One of the earliest studies, using two oral contraceptives containing the same amount of EE (35 mcg), and an unvaried dose of norethindrone in a monophasic pill and a threephase increase in norethindrone in the triphasic pill, found that monophasic users reported less vaginal lubrication than nonusers, whereas triphasic users did not [35]. Vaginal dryness was also a side effect of COC reported in a more recent prospective study, with 280 women randomized in three groups: (i) taking COC 20 mcg EE/100 mcg LNG, (ii) COC 15mcg EE/60 mcg gestodene, and (iii) vaginal ring users (15 mcg EE/120 mcg etonogestrel) [29]. And, overall, a negative influence on sexual desire was reported by the COC groups. A similar result was observed in a randomized trial of a monophasic (35 mcg EE/0.250 mg norgestimate) and a triphasic pill (35 mcg EE/0.180 mg, 0.215 mg, 0.250 mg norgestimate), from the Kinsey Institute, which assessed pre-COC use characteristics at baseline and regularly assessed women with the aim to predict acceptability and continuation of the pill when used for contraceptive purposes. Of the subjects who discontinued COCs or switched pills, 8% did so because of sexual side effects [36]. A statistically significant decrease in frequency of intercourse and psychosexual arousability was reported. These studies revealed that disturbances of sexual intercourse are crucial points for acceptability and compliance [29, 36].

There are quite a few trials that have demonstrated a negative impact of COCs on female libido. In a cross-sectional questionnaire study conducted among 349 sexually active community-based women, aged 20-65 years, the authors found that COCs users had significantly lower frequencies of sexual thoughts, interest and days of sexual activity per month, compared to nonusers [37]. A large study assessing female sexual function and contraception in over one thousand German medical students, found that COC users had lower scores on desire and arousal domains, as well as statistically significant lower total Female Sexual Function Index (FSFI) scores [38, 39]. (The FSFI is a 19-question tool covering six domains assessing desire, arousal, orgasm, lubrication, pain and satisfaction. Each domain is associated with a maximum score of up to six points out of the total FSFI score (maximum total score on all domains is 36 points) [40]. The lower the FSFI score, the higher the likelihood of sexual dysfunction.) As in other studies referenced earlier, once again, no correlation was found between specific types of COCs (androgenic or antiandrogenic progestin content, or different dosages of EE) and negative libido [39]. This absent association between a lower FSFI score in COC users, and the progestin androgenicity and/or the EE dose, was also observed in a large cohort of 2612 young university women [41].

Furthermore, as already discussed, despite the fact that COCs decrease bioavailable androgens, several reports have shown that this decrease does not imply a negative impact on libido [17, 42, 43]. COC users have reported higher frequency of sexual thoughts and interest compared to nonusers [35]. It is also important to reinforce that newer progestogens like DRSP have been linked to an improvement in sexual arousal and enjoyment, orgasm frequency, and satisfaction with sexual activity [44, 45]. These results are consistent with the already mentioned randomized, double-blind, controlled trial reporting that DRSP did not have an unfavourable effect on libido [27]. In a multicentre study the majority of women experienced no change in libido, comparing two COCs with 30 mcg EE: (i) association with DRSP and (ii) association with LNG, though, in both groups, a small percentage of women reported higher or lower libido compared with their normal experience [46]. Another study randomized 115 women to conventional vs. extended cycle with 20 mcg EE and 3 mg DRSP, where both groups suffer improvement in several sexual parametres [47]. Findings suggest that women who choose a continued-regimen OC should not expect a decrease in sexual functioning as a result [47, 48].

COCs have been associated with dyspareunia not only because of possible vaginal dryness, but also because of the risk of vestibulitis. COC use as a risk factor for provoked vestibulodynia is another divisive finding in the literature. Several studies point for an association between COC use and vestibular pain [49–53]. The likelihood of superficial pain during intercourse seems to be higher when OCs are first used at a young age and increase with duration of use [50, 52]. However, based on quantitative genital sensory analysis, no evidence was found that 20 mcg COCs adversely affect clitoral or vestibular sensitivity [54]. Other authors also reported that COC use was not a risk factor for vestibulodynia [55].

Regarding the associations with E2 component in COCs, there are some interesting studies where some positive sexual aspects may arise from the use of these pills. A preliminary study in 57 healthy women showed that E2V/DNG improved sexual enjoyment, arousal, orgasm, and desire over six cycles of use; yet, this study was open label, and without a comparator [56]. Later, a multicenter, randomized, double-blind study compared the effects of six cycles of E2V/DNG with EE/LNG on sexual function, in 276 women with COC-associated sexual dysfunction. Equivalent improvements in all domains of the FSFI were demonstrated in both groups, with no significant diferences [57]. Overall, these studies suggest that E2V/DNG does not have a negative impact on sexual function.

A recent prospective observational study focused on the effect of E2/NOMAC to improve the sexual function of women with low sexual desire due to COC usage. At baseline, the total FSFI score and the Female Sexual Distress Scale (FSDS) score was measured, both indicating sexual dysfunction with sexual distress. At the third (first follow-up) and sixth (second follow-up) COC intake cycles of E2/NOMAC pill, FSFI score was consistently higher and FSDS score continued to drop. Interestingly, the previous COCs that women were using, containing 30 mcg EE and DRSP 3 mg, or chlormadinone acetate 2 mg, or dienogest 2 mg, and 20 mcg EE and DRSP 3 mg, were all progestogens with antiandrogenic activity. However, there was no comparative COC group in the study [58]. And the common assumption that COCs containing antiandrogenic progestins have a detrimental effect on sexual function relative to those containing androgenic progestins is not irrefutable, quite the contrary [27, 57].

*(2) Progestin-Only Pills*. Progestin-only methods include pills and the most commonly used POP in Europe contains low doses of desogestrel. A placebo-controlled, double-blind comparison of COCs and POPs users was carried out in two contrasting cultures (Manila, Philippines, and Edinburgh, Scotland). All women had been sterilised or partners had been vasectomised. The POP was associated with no adverse impact on female sexuality in both centres [59].

#### 3.2.2. Contraceptive Ring, Transdermal Contraceptive Patch, and Injectable Contraceptive

Other widely used SARC methods are the vaginal ring (delivers 15 mcg EE per day) and the patch (delivers 20 mcg EE per day). The ring contains etonogestrel and the patch contains norelgestromin as their progestin component. The injectable contraceptive depot medroxyprogesterone acetate (DMPA) is a progesterone-only contraceptive method.

The effects of these other forms of hormonal contraception on sex drive have not been studied as comprehensively as OCs. As with COCs, there are conflicting results. In a randomized study, the better results related to desire and sexual satisfaction were obtained by vaginal ring users versus OC users (COC 20 mcg EE/100 mcg LNG and COC 15 mcg EE/60 mcg gestodene) [29]. Improvement of sexual desire was also noted in women using the contraceptive ring compared with a desogestrel-containing combined OC and a desogestrel-only OC [60]. A small prospective study found that women using the vaginal ring in an extended manner experienced improvements in sexual function and reduced sexual distress after 60 days [61]. On the contrary, an open-label randomized trial compared the contraceptive ring vs. COC (30 mcg EE + 3 mg DRSP) and observed that a decreased libido was more common with the ring [62].

Sexual function of first-time users of the contraceptive ring and contraceptive patch, who had recently used COCs, was assessed by FSFI scores in a multicentre randomized study. Slight decrements in sexual function scores were noted with contraceptive ring use and in several sexual domains, whereas slight increases were noted with patch use. However, authors concluded that these changes are not likely to be clinically significant [63].

Recently, a prospective cohort study, including data from 1983 women aged 14 to 45 years, found less interest in sex in women using the estrogen-progestin vaginal ring and DMPA, compared to nonhormonal contraception (copper IUD) use. A neutral effect on sexual desire was found in women using estrogen-progestin patch [64]. DMPA association with a lower sexual function was also supported by users who reported not finding sex pleasurable, compared to copper IUD users, and feeling anxious before sex, but this last difference was not statistically significant [64]. Nelson had also reported that 5.8% of women using DMPA have negative complaints on libido [65].

In contrast, no association was found between the use of DMPA injection and sexual interest among adolescent users when comparing various hormonal contraceptives [66]. Another study that evaluated OC vs. injectable progestin in their effect on sexual behavior found that COC users had lower levels of FT compared with DMPA users, but they were not different in sexual function [67]. Similarly, no difference was found in sexual interest of an adolescent population comparing DMPA users to nonusers of any hormonal method of contraception [68].

### 3.3. Long-Acting Reversible Contraception

LARC methods include LNG and copper IUDs, and the etonogestrel subdermal implants. LARC provides at least three years of continuous pregnancy protection and is highly effective (>99%) because it is not subject to errors in use that often reduce effectiveness of SARC [69].

In most studies, hormonal IUD did not alter sexual functioning, but some women who used an IUD had increased sexual desire or an overall improvement in their sex life. In a study of 200 women using the levonorgestrel-releasing intrauterine system (LNG-IUD), a significant beneficial effect of this method on sexual functioning (sexual desire and arousal) was found, compared to control groups of women using a different type of IUD and with women using no contraception [70]. Other studies have also reported high rates of satisfaction with both IUDs, hormonal and copper, but no difference in sexual function [64, 71].

Available evidence on the sexual effects of the hormonal IUD is based on the LNG-IUD with total content of 52 mg (20 mcg / 24 h). Until new data, it seems plausible to extrapolate these results to the newest IUD containing 13.5 mg of LNG.

Data on contraceptive implants and sexual health are more inconclusive. A lack of interest in sex was established in women using the progestin implant versus the use of a nonhormonal contraceptive [64]. Gezgine et al. found that 2.5% of women had the implant removed due to decrease of libido [72]. Anorgasmia after etonogestrel implant insertion as a possible rare adverse event was reported, quickly reversible with implant removal [73].

However, as already mentioned, nonoral hormonal contraception increased the positive indicators of female sexual function in a number of studies [29, 60, 61]. Guida et al. randomly assigned three groups of women to the contraceptive ring, COC (20 mcg EE/150 mcg desogestrel), or a control group. Women in both the ring and the COC group reported a global improvement of sexual function compared to women not using hormonal contraception [74]. These three groups were later compared with a new group of women, treated with a subdermal hormonal contraceptive containing 68 mg etonogestrel. A significant increase in the frequency of sexual intercourse, personal initiative, numbers of orgasm, and satisfaction has been observed in the group using subdermal hormonal contraception, compared to all other groups. As authors recognized, the major limitation of the study is that patients have been enrolled in two different periods. Nevertheless, all hormonal contraceptives tested seemed to have a positive effect on some aspects of sexual function in the users. The authors also concluded that subdermal hormonal contraceptive is better for women's sex life but its effect is slower compared to other hormonal contraceptives, because the sexual increments were effective only after six months [75]. In the same way, another study of the etonogestrel implant showed no negative effects on libido and on female sexual function [76].

## 4. Important Considerations about Sexual Health of Hormonal Contraception Users

Prior to starting hormonal contraception, it is necessary to evaluate the patient for contraindications and risks associated with this group of contraceptives. Based on this assessment and according to woman's preference, a method is recommended. It is also relevant to consider the impact that various hormonal contraceptives may have on sexual function when prescribing these methods to any woman. In fact, an assessment of sexual problems should be a part of every comprehensive woman's health visit. Women who discussed their sexual concerns with their clinician found the discussion helpful. In a questionnaire study of over 1000 women seen for a primary care visit, 98% reported at least one sexual complaint and only 18% of clinicians asked about their sexual health [77].

The World Health Organization (WHO) and the United States' Centers for Disease Control and Prevention (CDC) guidelines on contraceptive use do not mention the potential effects of hormonal contraceptives on women's sexuality [78, 79]. Yet, screening patients for preexisting FSD and informing them of the possible sexual side effects of hormonal contraception are important [43]. In the largest United States study of FSD, Prevalence of Female Sexual Problems Associated with Distress and Determinants of Treatment Seeking (PRESIDE), among approximately 3000 women identified with a distressing sexual problem, only 6% scheduled a medical visit specifically for a sexual complaint [6].

The mixed results on the interaction between the use of hormonal contraception and female sexual function do not allow absolute recommendations. However, possible sexual side effects require that they be considered and, when present, a therapeutic approach should be initiated.

As here reviewed, COCs with association of DRSP/EE, or gestodene/EE and LNG/EE, seem to be good choices for women with sexual issues because FSD symptoms are less likely to occur [27, 30, 44, 45, 47]. In daily clinical practice, the choice of a progestogen with a more androgenic action to enhance female sexual function seems to be a commonsense choice rather than an evidence-based one. In this sense, studies also suggest that women with COC-associated FSD can improve several sexual domains after switching to a combined E2V/DNG pill or to a COC containing E2/NOMAC [56–58].

It is also true that concerns about the different dosages of EE or about the elimination of the hormone-free interval in modern oral contraceptives as modulatory factors of female sexual function seem negligible [27–30, 47, 48].

Available evidence on the sexual impact of the hormonal LARC methods seems reassuring, especially LNG-IUD [64, 70, 71]. Hormonal IUD presents as a possible first-line alternative method of hormonal contraception to women who experience FSD with SARCs and do not desire fertility in a near future.

Sexual domains should not only be evaluated before starting hormonal contraception, but they should also be reviewed regularly. Physicians should ask open-ended questions regarding sexual health, rather than wait for women to initiate the conversation [80]. If left untreated, sexual problems can have a negative impact on the quality of life, self-esteem, and interpersonal relationships of these women.

A multidisciplinary approach, involving trained specialists and psychologists, is important when FSD is diagnosed. Conversely, hormonal contraception-related sexual impairment, when early detected, will allow these women to maintain reliable contraceptive choices, by having an informed conversation with the physician and discussing other options, as the switch of formulations or the route of administration. Changes in desire and sexual satisfaction during hormonal contraception are important elements that might relate to acceptability, compliance, and continuation of effective contraception [29, 36].

## 5. Concluding Remarks

Sexual specific effects of hormonal contraceptives are not well studied. The literature shows significant disagreement among studies on this topic, mainly because different methodologies are used. Only a few reports have attempted to address this issue amid immense scientific research into the adverse effects of hormonal contraception. And most of those who addressed sexual dysfunction in women did not assess whether sexual issues are associated with personal distress, a key finding for the diagnosis of FSD.

This review suggests that hormonal contraception can cause female sexual response impairment. The how and to whom are open critical questions. Estrogens and androgens play a role in female sexual function, but the magnitude of their effects needs further investigation. Several studies have linked hormonal contraception to negative effects of sexual function, but also to a neutral effect, or an improvement in the sexual domains, compared to women who do not use hormonal methods or who do not use any type of contraception.

The multifaceted nature of female sexual function shows the importance of ascertaining of a temporal relationship between the onset of sexual complaints and initiation of a hormonal contraceptive. There are so many factors that affect the sexual response that the positive or negative impact attributable to hormonal contraception can be covered. On the other hand, the diagnosis of FSD requires a holistic assessment where the use of a hormonal contraceptive can sometimes be mistakenly regarded as the perfect suspect.

It is also clearly possible that the sexual behavioral impact of exogenous administration of progestogens and/or estrogens varies in women, which would explain that negative or positive effects are restricted to subgroups. It is not yet possible to predict which women are likely to experience adverse effects of hormonal contraception on their sexuality, nor which oral formulations or nonoral routes of administration are most likely to be responsible.

It is important to highlight the individuality of each woman's sexual health, increasing the complexity of this field. In order to improve compliance with hormonal contraceptives, it seems appropriate to incorporate women's sexual health into contraceptive counseling and to maintain the assessment of sexual function as an integral part of follow-up consultations. Hormonal contraception is a milestone in women's health and any approach to maximizing its benefits should be a priority.

## Abbreviations and Acronyms

CDC: Centers for Disease Control and Prevention
COC: Combined oral contraceptive
DMPA: Depot medroxyprogesterone acetate
DRSP: Drospirenone
E2: Estradiol
EE: Ethinylestradiol
E2/NOMAC: 17*β*estradiol and nomegestrol acetate
E2V/DNG: Estradiol valerate and dienogest
FDA: Food and Drug Administration
FSD: Female sexual dysfunction
FSH: Follicle-stimulating hormone
FT: Free testosterone
HPO: Hypothalamic-pituitary-ovarian
IUD: Intrauterine device
LARC: Long-acting reversible contraception
LH: Luteinizing hormone
LNG: Levonorgestrel
LNG-IUD: Levonorgestrel-releasing intrauterine system
OC: Oral contraceptive
POP: Progestin-only pill
SARC: Short-acting reversible contraception
SHBG: Sex hormone-binding globulin
WHO: World Health Organization.
